# Impact of Washing Parameters on Thermal Characteristics and Appearance of Proban^®^—Flame Retardant Material

**DOI:** 10.3390/ma15155373

**Published:** 2022-08-04

**Authors:** Tea Kaurin, Tanja Pušić, Tihana Dekanić, Sandra Flinčec Grgac

**Affiliations:** Faculty of Textile Technology, University of Zagreb, 10000 Zagreb, Croatia

**Keywords:** cotton fabric, Proban^®^, environmentally friendly washing procedure, flame retardant durability, MCC, appearance

## Abstract

Proban^®^ is a multiphase treatment of cotton fabrics based on the formation of pre-condensates using the flame retardant (FR) agent tetrakis (hydroxymethyl) phosphonium salts (THPx). The assessment of the durability of a product demands a preliminary understanding of how relevant it is to extend its lifetime. It is therefore important to minimize the risk of agents impacting: (1) the protection level, (2) shape and dimensions, and (3) additional comfort characteristics of the fabric. This research focused on the impact of washing conditions on the durability of FR properties and appearance of Proban^®^ cotton fabrics, which was systematically arranged through the variation in the chemistry distribution in the Sinner’s circle. The chemical share was varied in laboratory conditions as a simulation of industrial washing based on component dosing, where the temperature, time and mechanical agitation were constant. The washing of cotton fabrics was performed through 10 cycles in four baths containing high alkali components, medium alkali components, high alkali reference detergent and water. The environmental acceptability of washing procedures through effluent analysis was assessed by physico–chemical and organic indicators. The limited oxygen index (LOI), calorimetric parameters (micro combustion calorimetry), thermal stability and evolved gases during thermal decomposition (thermogravimetric analyzer (TGA) coupled with an infrared spectrometer (TG–IR)), surface examination (FE-SEM), spectral characteristics and pH of the aqueous extract of the fabrics before and after 10 washing cycles were selected for proof of durability. The medium alkali bath was confirmed as a washing concept for Proban^®^ cotton fabric through the preservation of FR properties examined through LOI, TGA, TG–IR and MCC parameters and appearance color and low level of fibrillation.

## 1. Introduction

Extreme operating conditions of exposure to the effects of fire, fuel explosions or heat radiation require the use of appropriate protective clothing systems, covering gloves, hoods, upper layers of clothing and appropriate footwear [1]. Particular attention should be paid to the selection of materials that contribute to the comfort and safety of clothes. For this reason, such clothing must, in addition to the protection requirement, meet a number of other requirements, by which it is classified as comfortable and safe to human health and the environment [2,3].

In order to protect humans in open flame fires and extreme heats, the fabric is significant in the preservation and protection of the human body [4].

The composition and structure of the material, its technical characteristics, degree of pre-treatment, finishing and external environment conditions (weather, heat, air flow, oxygen concentration) influence the material’s flame retardancy [5]. Cotton is often used because of its high comfort characteristics, but it is easily flammable and generates burns [4]. Various treatments attempt to quench the flames, reduce the combustible gases and strengthen the generation of non-combustible gaseous degradation products in order to achieve flame retardancy. Most cellulose materials are non-durable or semi-durable and as such have limited lifetime [1]. The design of more durable products is the key strategy to preserve material properties and reduce the waste amount [6].

Proban^®^ treatment, introduced by Albright & Wilson from England, whose protective rights and licenses were later transferred to the company Rhodia, is based on the formation of pre-condensates when using a FR agent based on tetrakis (hydroxymethyl) phosphonium salts (THPx) in multiphase treatments. The first is pre-condensation of tetrakis-hydroxymethyl chloride (THPC) or sulphate (THPS) with urea. Prior to application onto cotton, the pH value of the pre-condensate solution is adjusted to 6.0, thus preventing the settling of insoluble products due to passing a higher content of phosphonium salt to the form of phosphine [1]. If the pH rises above pH 6.0, the pre-condensate reactivity decreases due to the formation of phosphine oxide.

Polymerization in the Proban^®^ process is based on the application of gaseous ammonia at room temperature. During the application and thermocondensation process, resins with six reactive P-methylol groups are formed that react with each other [1,7]. The central phosphorous atom in the P-methylol groups may be in one of three states (phosphonium, phosphine and phosphine oxide).

After impregnation, the fabrics are dried and treated with gaseous ammonia in a specially designed chamber where crosslinking takes place. In this phase, phosphorus is still in a lower (III) oxidation state (phosphine oxide). The fabric is treated with an aqueous solution of hydrogen peroxide for oxidation. The key factor is that the final product does not have hydrolyzed bonds on phosphorus, which is completely in a stable phosphine oxide structure. The efficiency of FR cellulose materials depends on process parameters in the domain of technological and use durability or lifetime, so Proban^®^ should be considered a material whose FR properties can be weakened after several washing cycles [4,5,6]. The assessment of the durability of a product can be based on two aspects, technical and usage (e.g., duration of use, supplier and consumer requirement), as well as on other environmental and economic aspects (e.g., life cycle, cost of products) [4,8,9].

In many cases, some parameters of the Sinner’s circle [10], e.g., high washing temperatures, excessive amount of detergents and other treatments to remove soils, stains and odor can impair the properties of personal protective clothing. Washing is a complex process involving physical and chemical parameters: temperature, chemistry (types, composition and amount), time (length of a process) and applied mechanical agitation [11,12]. Parameters depend on each other, so a reduction in one factor has to be compensated by an increase in the others.

Water as a washing medium is the fifth parameter of the Sinner’s circle. Detergents contain bleach, fragrances, softeners and other additives that may leave residues on the surface of textiles [13]. Changes in surface properties and pore volume are the main causes of the change in material properties. It is therefore important to minimize the risk of agents impacting the protection level, shape, dimensions and comfort characteristics [14].

The present research focuses on the chemistry as a Sinner’s circle parameter to verify the concept of a lower alkalinity washing bath compared to conventional, standard detergent and water, through the assessment of functional properties and appearance, and all guided by the idea of product research according to product life cycle assessment criteria. The limited oxygen index (LOI), calorimetric parameters (MCC), thermal gravimetric analysis (TGA), coupled thermal gravimetry–Fourier transform infrared technique (TG–FTIR), surface examination (SEM), spectral characteristics and pH of an aqueous extract of fabrics before and after washing were selected as criteria for proof of the concept. Future research will investigate the environmental acceptability of washing procedures through effluent analysis to gain complete knowledge of environmental impacts.

## 2. Materials and Methods

### 2.1. Material

Research was conducted on a FR functional cotton fabric specified as a Proban^®^ described in Table 1.

### 2.2. Washing Procedures

The textile material was subjected to 10 washing cycles under different conditions specified in Table 2.

In the washing process, the fabric labelled as UNW was treated in Wascator FOM71 CLS, (Electrolux, Stockholm, Sweden) for 10 cycles. The component dosage system was used in two variations (46_10x and 47_10x), differing in alkalinity, where 46_10x represented higher alkalinity compared to 47_10x as lower alkalinity. The washing process (49_10x) continued with standard ECE-1 detergent and water (49_10x). All washing protocols are technically specified in Table 3.

The discharge from the laboratory washing machine after protocols specified in Table 3 were collected and analyzed by standard methods.

### 2.3. Methods

Different methods were selected for the characterization of the washing baths and effluents as well as fabrics before and after the washing protocols specified in Table 3.

The pH measurement of the washing baths (46, 47, 49), which was taken directly from the machine without fabrics was determined using the pH meter Mettrel.

Physico–chemical characteristics of collected washing effluents (46, 47, 48 and 49) were monitored by determining pH value, conductivity (κ), turbidity (T), total dissolved solids (TDS), total suspended solids (TSS), chemical oxygen demand (COD) using standard analytical methods.

The characterization of fabrics before and after 10 cycles of the washing protocol was based on:

1. residual substances as pH of the aqueous extract [ISO 3071: 2020].
2. limiting oxygen index (LOI).
3. microscale combustion calorimetry (MCC).
4. thermal stability and evolved gases during thermal decomposition (thermogravimetric analyzer (TGA) coupled with an infrared spectrometer (TG–IR).
5. microscopy, SEM and digital.
6. spectral characteristics.

The limiting oxygen index (LOI) technique was used for assessing the ease of ignition as an important parameter for the characterization of FR textile materials. The LOI values of the materials were determined according to ASTM D2863-10 and presented the maximum percentage of oxygen [O_2_] in an oxygen-nitrogen gas mixture [O_2_] + [N_2_] that will sustain burning a standard sample for a certain time. The LOI values were calculated according to Equation (1).

(1) $$LOI=\frac{\left[O_{2}\right]}{\left[O_{2}\right]+\left[N_{2}\right]}\times 100\;\left[\%\right]$$

The microscale combustion calorimeter (MCC) was used for the thermal characterization of FR samples before and after washing. The measurement was performed using an MCC-2 micro-scale combustion calorimeter (Govmark, Farmingdale, NY, USA) according to ASTM D7309-2007. The sample of 5 mg was heated to a specified temperature using a linear heating rate of 1 °C/s in a stream of nitrogen, with a flow rate of 80 cm^3^/min. The thermal degradation products were mixed with a 20 cm^3^/min stream of oxygen. Each sample was run in triplicates, and the presented MCC parameters are the averages of the three measurements.

The thermogravimetric analysis (TGA) was carried out using a Pyris 1 TGA (Perkin Elmer, Waltham, MA, USA). The sample weight was adjusted to 5–6 mg and the experiment was conducted in air atmosphere. All samples for the TGA were analyzed in the temperature range from 30 °C to 850 °C in a continuous air or nitrogen flow. The temperature was increased at a rate of 30 °C/min.

The TGA was connected to the Fourier transform infrared spectrometer (FT–IR, PerkinElmer, Spectrum 100S, Shelton, CT, USA) with a TG–IR interface. Evolution profiles of different compounds were tracked by FT–IR. The combination of TGA with FT–IR allowed for the analysis of the nature of the gaseous products formed in TGA and their online monitoring. Nitrogen, which does not exhibit IR-absorption, was used as the purge gas. Air was used as the reaction gas, so the end products of the decomposition were pyrolysis rather than oxidative degraded products. The FT–IR spectrum were acquired throughout the run at a temporal resolution of 4 s and a spectral resolution of 4 cm^−1^. A thermal analysis gas station (TAGS), equipped with a detector, was used for the FT–IR analysis. The transfer line, high-temperature flow cell, and TG interface were held at 280 °C for the duration of the run to prevent sample condensation. The evolved gases were transferred through the FT–IR flow cell by a peristaltic pump with a flow rate of 60 cm^3^/min.

The field emission scanning electron microscope, FE-SEM Mira LMU, Tescan, Brno, Czech Republic, was used for the examination of samples at 20 kV. A digital microscope was also applied for surface examination.

The spectral characteristics were observed as the difference between the washed samples compared to the unwashed sample through: the total difference in color (dE), difference in lightness (dL*), difference in chromaticity (dC*) and difference in hue (dH*) as the average values of four measurements conducted on the remission spectrophotometer DC 3890(Datacolor, Dietlikon, Switzerland).

## 3. Results and Discussion

Analyses of the properties of the fabric after 10 cycles of the washing process in relation to the pristine fabric were carried out systematically as described in the experimental section. Proban^®^ fabrics were washed over 10 cycles under laboratory conditions according to different protocols: the component wash as a simulation of the industrial process (46_10x) specified as a high alkali and less alkali component wash (47_10x).

Protocols 48 and 49 underwent the same phases but differed in the chemical share in the Sinner’s circle of the washing process. Process 49 was carried out with a standard detergent and process 48 in water only. It is known that through the interaction with textiles water has an impact on the textiles’ properties [15]. The measurements of the pH washing bath protocols 46 and 47 were conducted without fabrics, Table 4.

The results in Table 4 show differences in the pH of the washing baths affected by the dosage of NaOH in the programs 46 and 47. The composition of standard detergent ECE-1 (program 49) reflects an alkali bath.

Results of washing effluents analysis through physico–chemical and organic indicators are presented in Table 5.

The values of physico–chemical characteristics of the collected effluents presented in Table 5 show the differences between particular programs. If pH and conductivity are considered, the differences are conditioned by the share of chemistry in the washing process. Component washing (programs 46 and 47) with the neutralization phase caused acidity of effluents (pH lower than 3.0). Washing programs 48 and 49, involving rinsing in water, resulted with higher pH (higher than 5.0). The small difference is obtained in the turbidity values, all effluents possess about 30 NTU. Negligible difference was obtained with total suspended matter (TSS) caused by fibrils released in all of the tested washing processes. The biggest difference is manifested in the load of effluent with chemical substances (COD), where the component washing programs according to the industrial concept (46 and 47) have ten times more values compared to washing with standard detergent and water. The characterization of fabric properties was carried out to verify whether a lower alkaline bath has better potential to preserve the functional properties and appearance of fabrics for a longer life cycle. As such, the residual alkali of fabrics was analyzed by measuring the pH of the aqueous extract, as shown in Table 6.

The pH values of the aqueous extract of untreated and washed fabrics indicate some differences: the pH value of samples washed in 46, 47 and 49 is slightly above 7.0. Fabrics washed in programs 46_10x (higher alkalinity) and 47_10x (lower alkalinity) were neutralized by an acid, so the impact of higher alkalinity on the pH of the aqueous extract is not so prominent.

An unexpectedly high pH value of the aqueous extract 48_10x can be attributed to the nature of substances released from a Proban^®^. The swelling ability of cotton in water (48) and the influence of the process parameters of the Sinner’s circle, expressed through mechanical agitation and temperature on the cellulose polymer structure, led to the increased alkalinity.

The limiting oxygen index (LOI) technique provides a quantitative measure for the determination of reduced flammability of the material, Table 7.

LOI values for all samples after 10 wash cycle regardless of the treatment conditions (46, 47, 48 and 49) retain the same value as the pristine Proban^®^ sample (32%). Such a high-retained value classifies all samples as self-extinguishing materials.

The MCC analysis results of the samples before and after washing are specified in Table 8 as: heat release capacity (ηc), maximum specific heat release rate (Q_max_), specific heat release (hc), specific heat of flammable gases (hc, g), temperature at maximum specified heat release rate (T_max_) and combustion residue.

The resulting values of the released heat capacity (ηc) presented in Table 6 show that the untreated Proban^®^ sample (UNW) has the lowest value, as expected. When considering the sample after 10 washing cycles in water (48_10x), it is obvious that the slight increase in the heat release capacity (ηc) compared to the pristine sample correlated with the resulting LOI value. The heat release capacity (ηc) of the samples 46_10x has the lowest value and the lowest specific heat release, hc, gas = 2.2 kJ/g compared to the other samples. An important parameter for monitoring the thermal stability of the samples is the pyrolysis residue. The sample washed in water (48_10x) had the largest residue—39.83%. Slightly less residue was found in the sample washed with a standard detergent containing phosphates (49_10x)—38.78%, while the smallest residue was found in the sample 47_10x—35.63%. The highest specific heat value of fuel gases was recorded for this last sample, probably due to the combustion of organic sample components.

**Figure 1:**
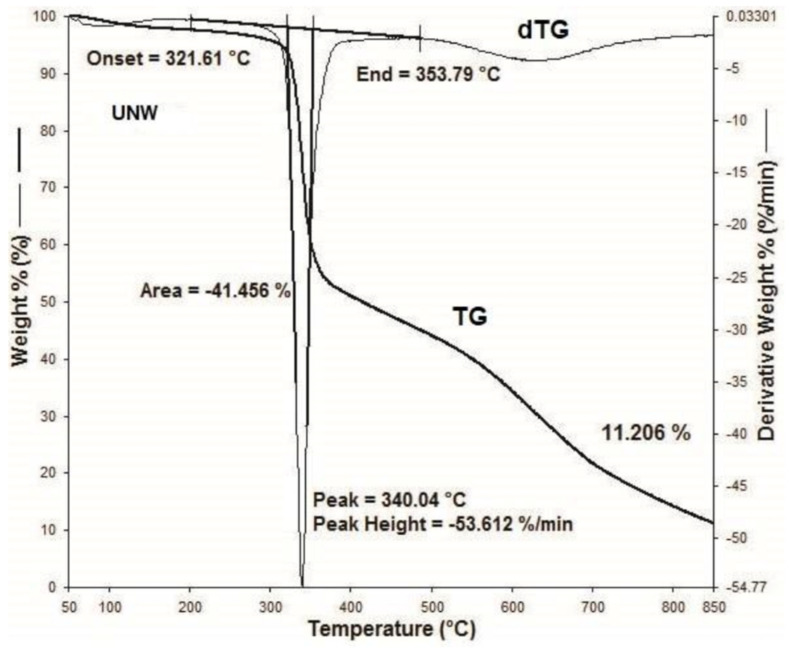
Thermogravimetric analysis of the untreated UNW sample: TG curve, dTG curve.

**Figure 2:**
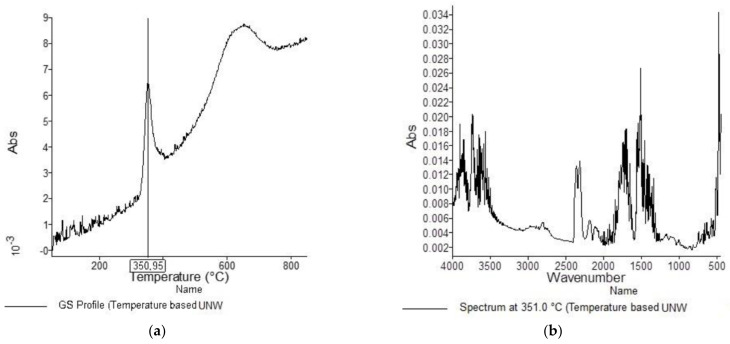

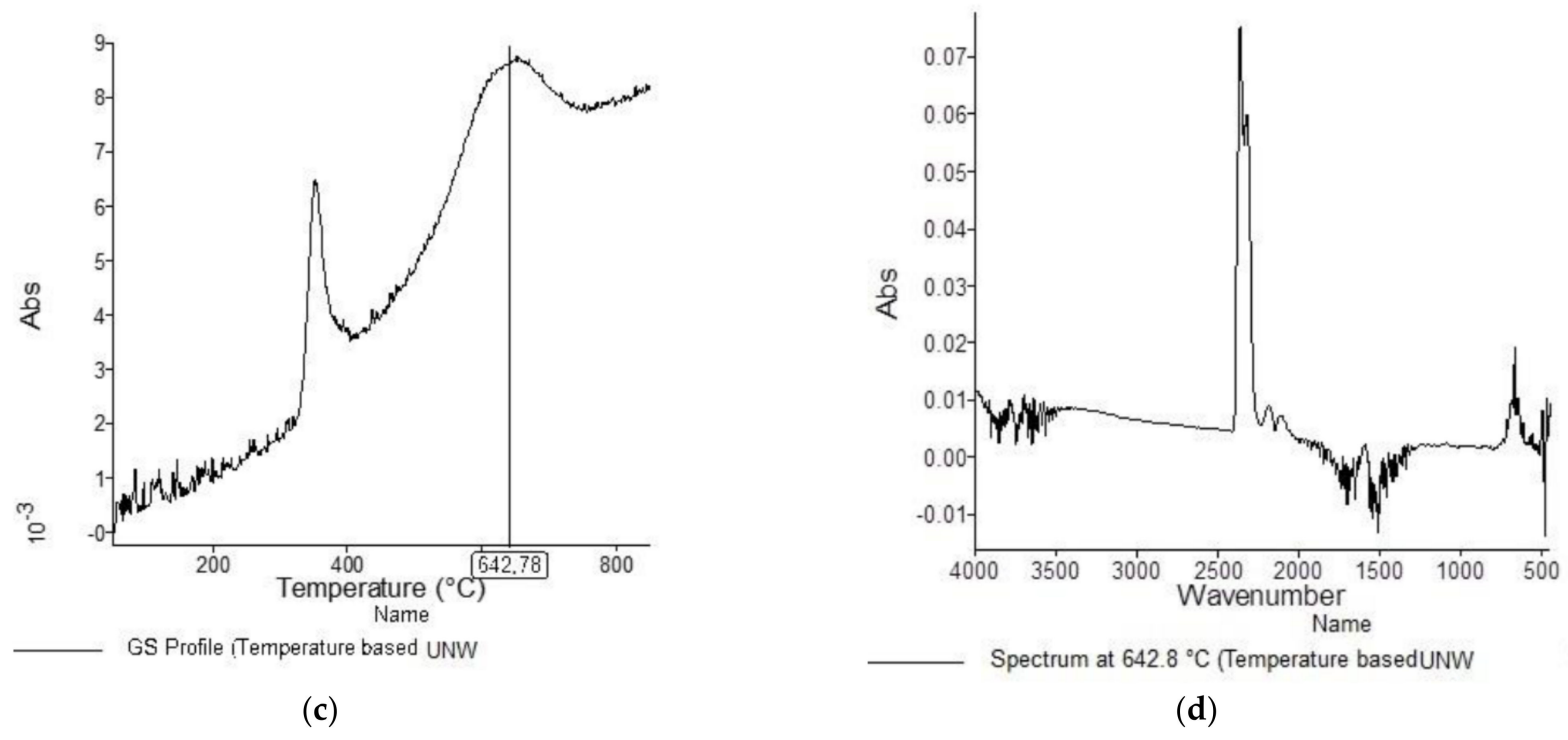
TG–IR analysis of cellulosic material treated with Proban^®^ method during thermooxidative decomposition: (**a**) and (**c**) intensity profile FT–IR spectrum of gas evolved, (**b**) FT–IR spectrum of gas evolved at 351.0 °C, (**d**) FT–IR spectrum of gas evolved at 642.8 °C.

**Figure 3:**
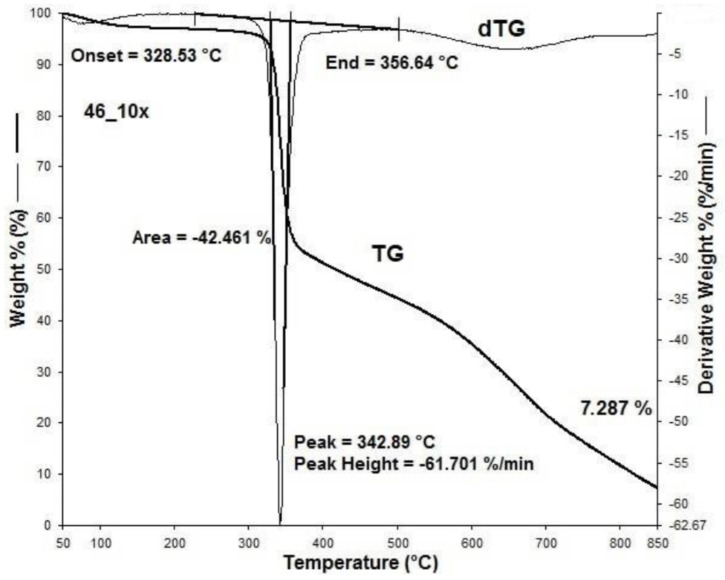
Thermogravimetric analysis of 46_10x sample: TG curve, dTG curve.

**Figure 4:**
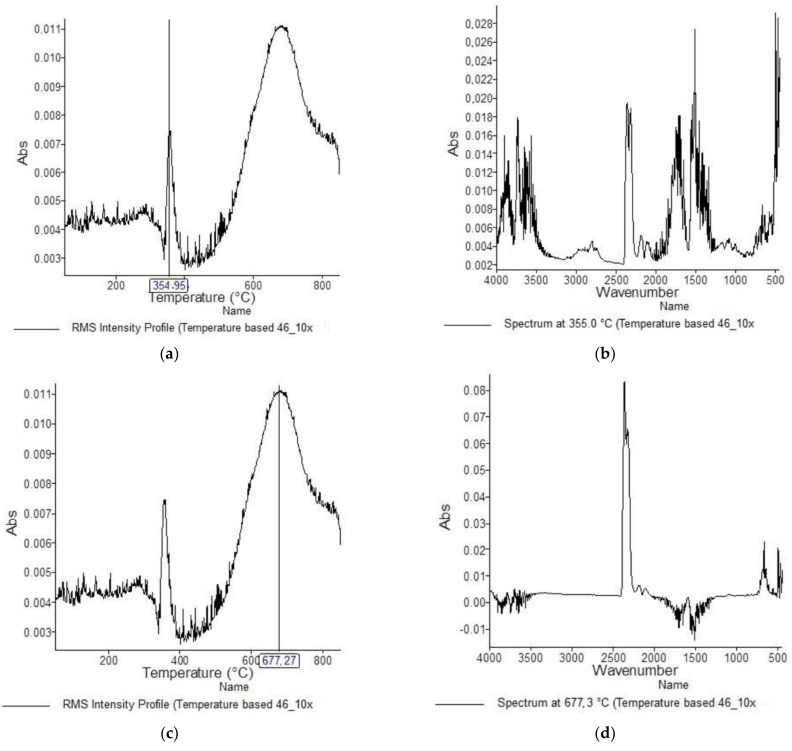
TG–IR analysis of 46_10x during thermo–oxidative decomposition: (**a**) and (**c**) intensity profile FT–IR spectrum of gas evolved, (**b**) FT–IR spectrum of gas evolved at 355.0 °C, (**d**) FT–IR spectrum of gas evolved at 677.3 °C.

**Figure 5:**
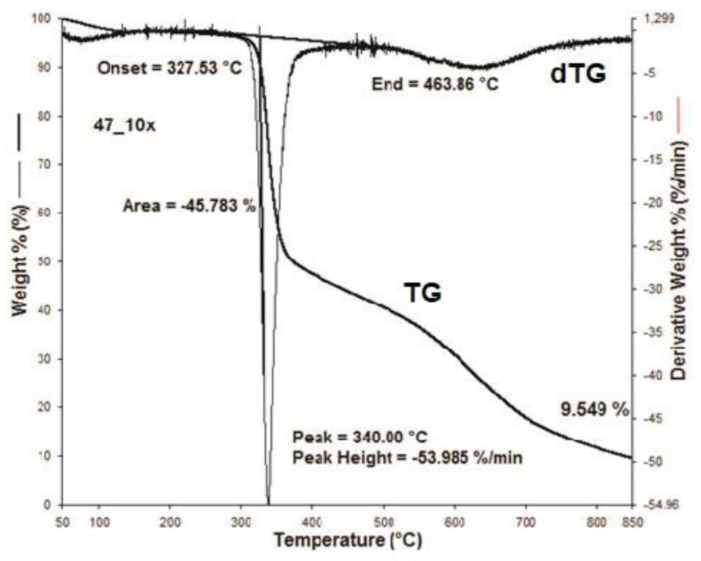
Thermogravimetric analysis of 47_10x sample: TG curve, dTG curve.

**Figure 6:**
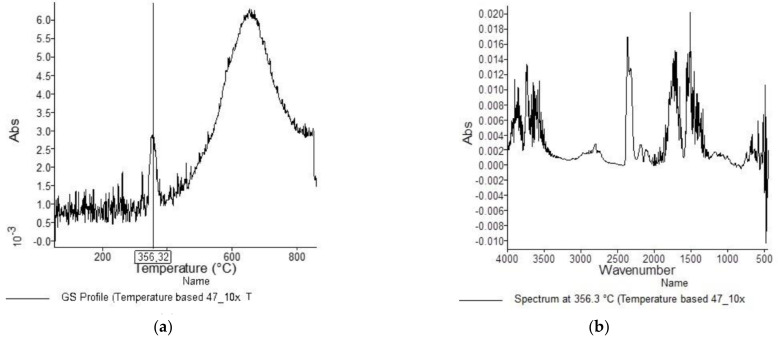

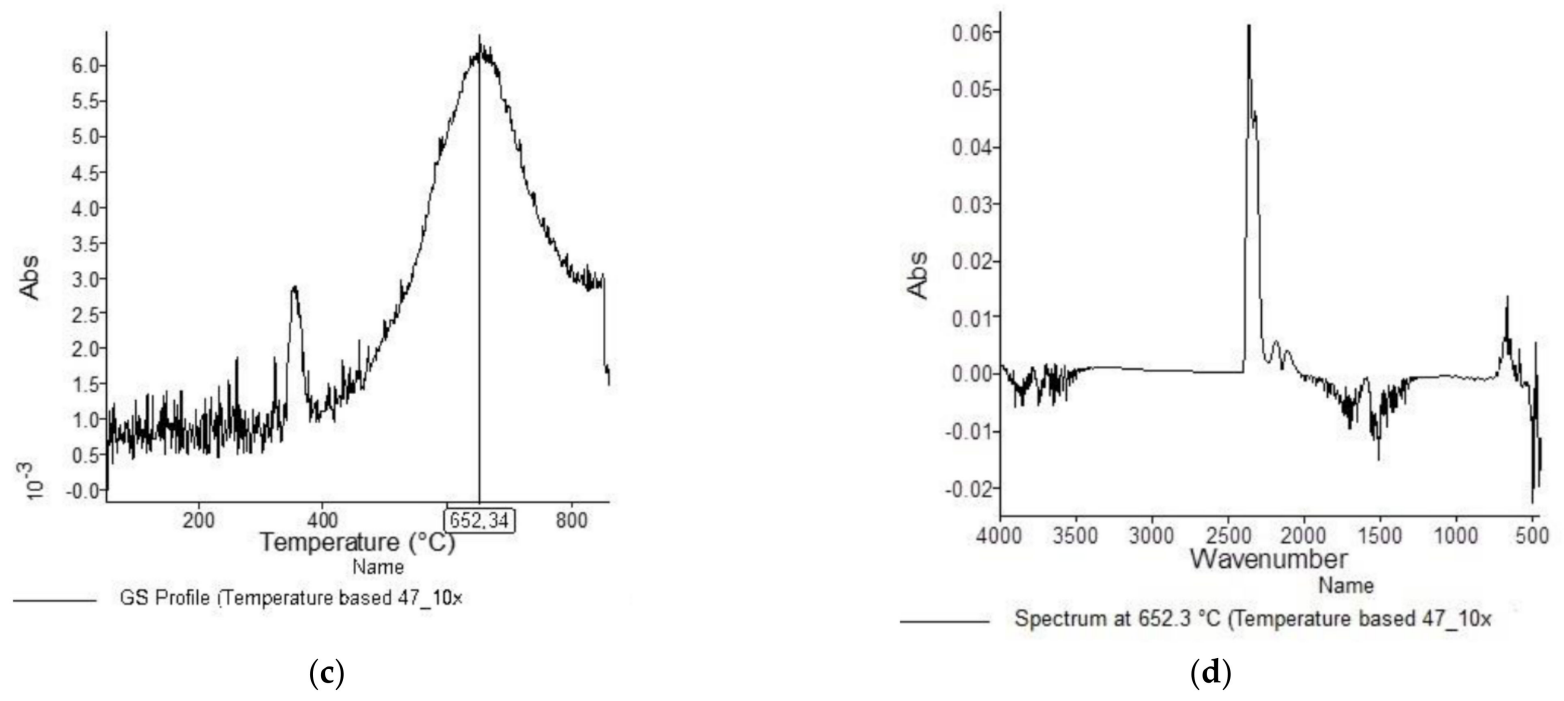
TG–IR analysis of 47_10x during thermo-oxidative decomposition: (**a**) and (**c**) intensity profile FT–IR spectrum of gas evolved, (**b**) FT–IR spectrum of gas evolved at 356.3 °C, (**d**) FT–IR spectrum of gas evolved at 652.3 °C.

**Figure 7:**
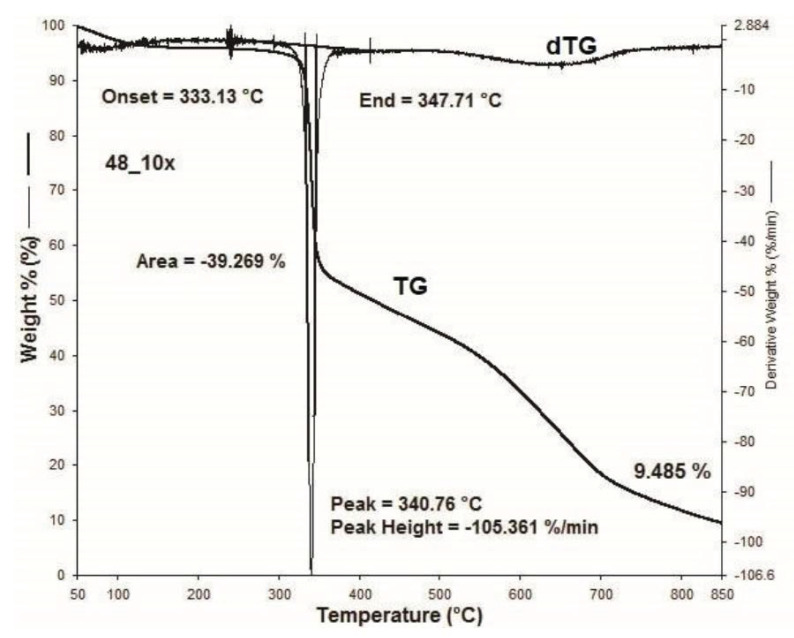
Thermogravimetric analysis of 48_10x sample: TG curve, dTG curve.

**Figure 8:**
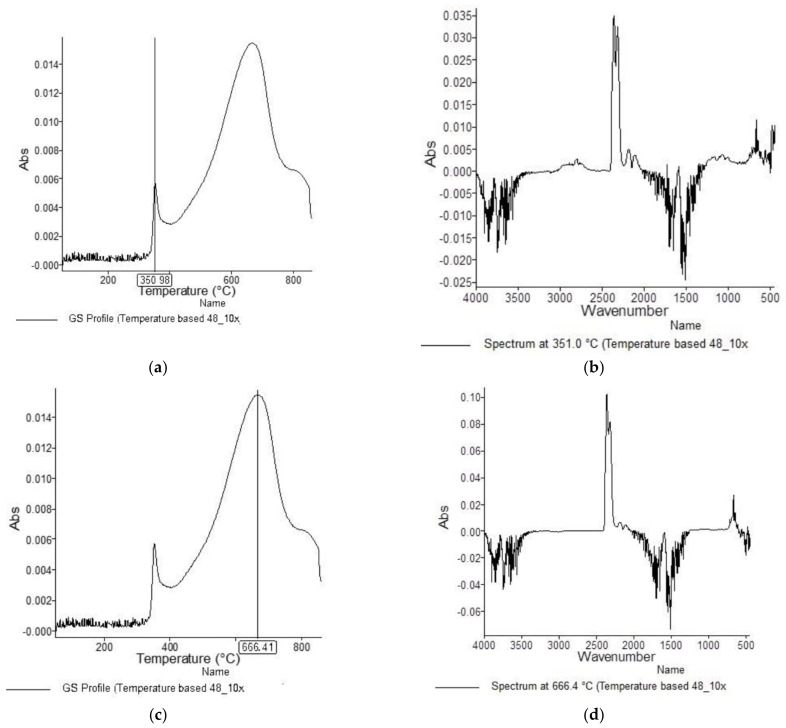
TG–IR analysis of 48_10x during thermo-oxidative decomposition: (**a**) and (**c**) intensity profile FT–IR spectrum of gas evolved, (**b**) FT–IR spectrum of gas evolved at 351.0 °C, (**d**) FT–IR spectrum of gas evolved at 666.4 °C.

**Figure 9:**
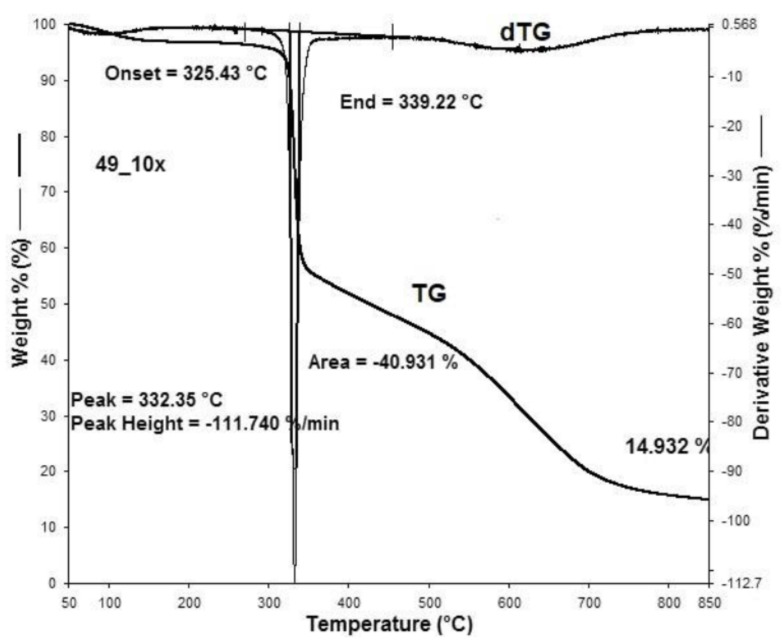
Thermogravimetric analysis of 49_10x sample: TG curve, dTG curve.

**Figure 10:**
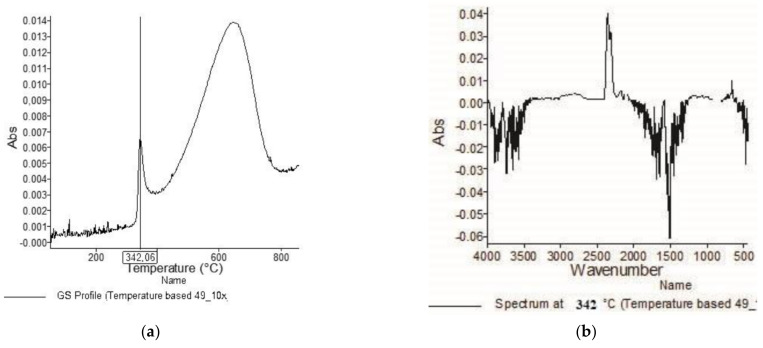

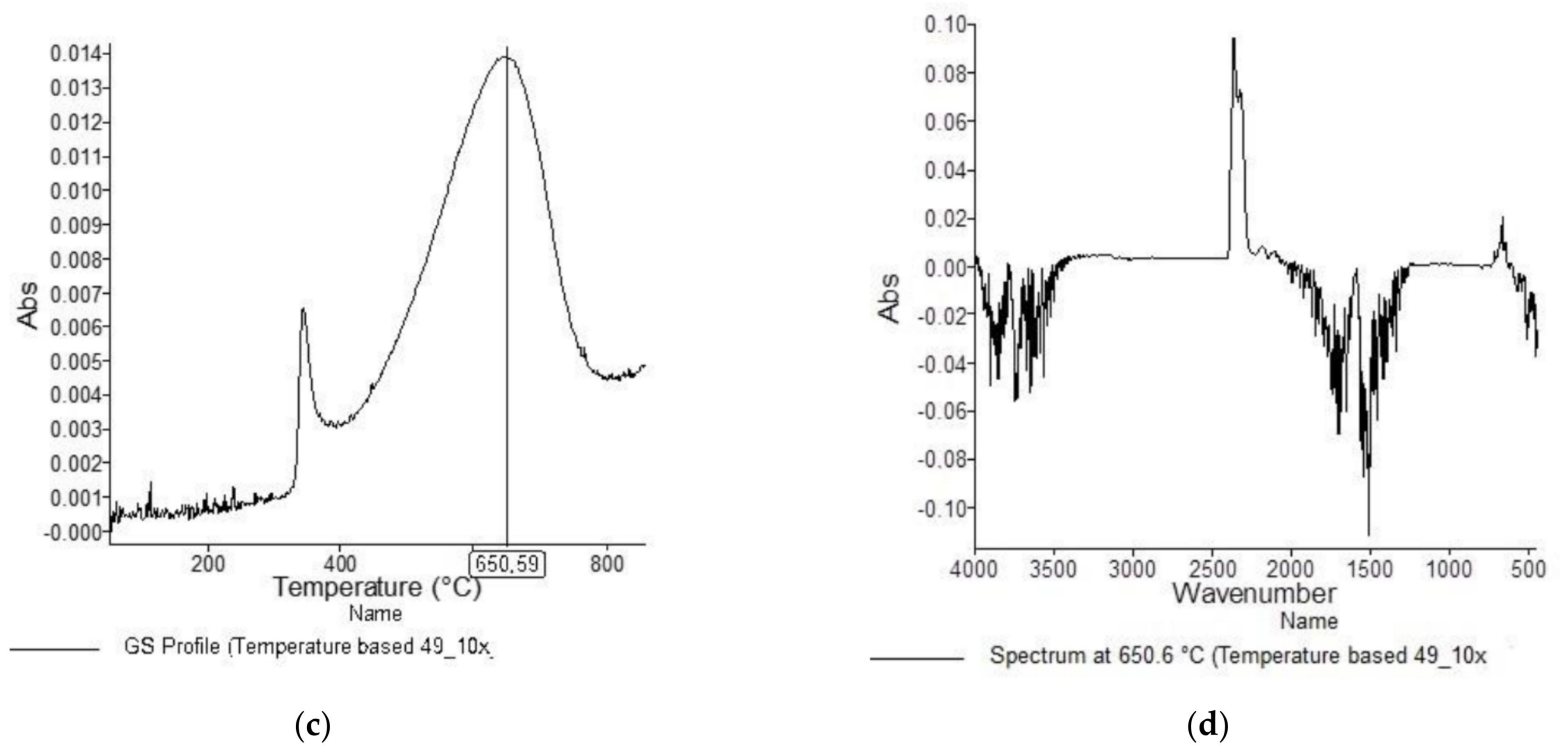
TG–IR analysis of 49_10x during thermo–oxidative decomposition: (**a**) and (**c**) intensity profile FT–IR spectrum of gas evolved, (**b**) FT–IR spectrum of gas evolved at 344.9 °C, (**d**) FT–IR spectrum of gas evolved at 650.6 °C.

Figure 1, Figure 2, Figure 3, Figure 4, Figure 5, Figure 6, Figure 7, Figure 8, Figure 9 and Figure 10 show the TG and dTG curves of all samples and the absorption spectrum of gaseous decomposition products formed during thermogravimetric analyses. All the tested samples showed similar properties during thermal decomposition in an air stream. From the first derivations of thermogravimetric curves (dTG) (Figure 1, Figure 3, Figure 5, Figure 7 and Figure 9) it can be seen that the dynamic degradation in all samples takes place in one step with the maximum speed of degradation at a temperature of about 340 °C, but differs in the onset and end temperatures of degradation and the residue at 850 °C. In order to determine the influence of 10 washing cycles on the thermal stability of the Proban^®^ sample, the thermal properties of the initial sample (UNW) were also examined. The dynamic thermal decomposition of the Proban^®^ sample (UNW) started at a temperature of 321.1 °C and ended at 353.79 °C. From the dTG curve it is evident that the degradation of the sample took place in one step with a mass loss of 41.456%. At the end of the dynamic decomposition, a higher intensity profile FT–IR spectrum of gas evolved was recorded, the composition and quantity of which were detected by IR. The characteristic peaks that appear in the range of 3500–4000 cm^−1^ and 1550–1566 cm^−1^ belong to the released water vapour. CO and CO_2_ peaks are visible in the area of 2179 and 2110 cm^−1^. Peaks belonging to the released CO_2_ were also detected in the range of 2359 and 2322 cm^−1^, while the occurrence of peaks at wave numbers 2951 and 1184 cm^−1^ indicates the release of RCHO [16]. From the absorption curves of the gaseous products of Figure 2b,d, it is apparent that a slightly higher amount of CO_2_ was recorded at the second intensity profile FT–IR spectrum of gas evolved (Figure 2c) at 643 °C, which is according to the TG curve at the remaining 30% of the mass. After that the gaseous products are insignificant or absent, which indicates the effect of the treatment aimed at suppressing the development of gaseous products during thermal decomposition. This is very important for monitoring the behavior of the sample in the open flame because it indicates the formation of charred residue and the sample becomes fireproof, which is confirmed by the high LOI value of 32 and low values of specific heat of gas decomposition products of 2.0 KJg^−1^ obtained by the MCC measurements. The best heat resistance results could be found in the sample washed with ECE standard detergent containing 30% phosphate (49_10x), which is visible from the residue at 850 °C, which is 14.932% (Figure 9). From the obtained absorption intensities of gaseous decomposition products (Figure 4a,b, Figure 6a,b and Figure 8a,b) it is obvious that in the samples 46_10x, 47_10x, 48_10x the first intensity profile FT–IR spectrum of gas evolved degree occurs at temperatures from 354 °C to 356 °C, while in the sample 49_10x it is at a slightly lower temperature of 345 °C, indicating an earlier onset of thermal decomposition, which is characteristic of more thermally stable materials. In all samples, gaseous products of CO_2_ and CO were recorded, but also the appearance of all the detected gases as in the UNW sample. The second degree intensity profile FT–IR spectrum of gases evolved are visible in Figure 4, Figure 6 and Figure 8, under the c. and d. ranges from 650 to 680 °C. The lowest temperature of the second absorption maximum of gaseous decomposition products was also recorded in the sample 49_10x and was 650 °C (Figure 10c), while the composition of gaseous products shown in Figure 10d indicates the presence of CO_2_ and CO with an absorption intensity of CO_2_ of 0.1. The comparison of the unwashed sample (UNW) and sample 49_10x clearly indicates that during the maintenance process there was an increase in thermal stability, proving a positive effect of the detergent. However, such applications are limited due to the known problems of phosphate impacting the ecosystem. The high amount of residue after the TG analysis in sample 49_10x was due to residual substances derived from detergent components that were not used to remove stains, but were deposited on the cotton fabric. Very similar behavior in terms of thermal stability and incombustibility to the sample 49_10x is shown by sample 47_10x, with the remark that the adsorption intensity of the decomposition gases recorded at the second maximum (indicating the presence of CO_2_—Figure 6c,d) is lower compared to the sample 49_10x and is 0.06, which suggests the possible application of such an environmentally friendly washing procedure in the maintenance of Proban^®^ samples.

The appearance of the fabrics before and after washing was examined by spectral parameters and microscopy, Table 9 and Table 10.

According to the results in Table 7, the spectral characteristics of blue cotton fabrics changed through 10 washing cycles, and the intensity of changes depended on the composition of the washing bath. The decrease in alkalinity (47_10x) proved to be the most favourable factor for the preservation of blue color characteristics.

The surface images of the washed samples, Table 8, indicate a specific fibrillation that can be caused by the characteristics of cotton cellulose and the parameters of the Sinner’s circle in the washing process. As the mechanical agitation and temperature were constant in the programs 46, 47, 48 and 49, the significant change in appearance specified for the fabric washed in the program 46 may be caused by the stronger swelling ability of cotton cellulose in alkali conditions of this washing bath.

## 4. Conclusions

The present research focused on the impact of washing conditions on the effluent composition and durability of FR properties and appearance of Proban^®^ cotton fabrics and was systematically arranged through the variation in the chemistry distribution in the Sinner’s circle. The chemical share was varied in laboratory conditions as a simulation of industrial washing based on component dosing, where the temperature, time and mechanical agitation were constant. The washing of cotton fabrics was performed through 10 cycles in four baths containing high alkali components, medium alkali components, high alkali reference detergent and water.

The results of effluent analysis showed negligible difference in the characteristics of high and medium alkali process. More optimal characteristics possess effluents from washing with standard detergent and water.

LOI as a parameter is not relevant for precisely determining mutual differences in FR properties, but is a valuable method for the rapid characterization of samples with respect to flame resistance. Proof of the medium alkali bath as a washing concept for Proban^®^ cotton fabric was confirmed through the preservation of FR properties examined through TGA and MCC parameters and appearance based on preserved color and a low fibrillation level. The proposed concept of medium alkali washing can be considered environmentally friendly since it shows the lowest intensity of the gases evolved and economically viable compared to other washing processes in this research, which is in line with LCA product monitoring guidelines.

Finally, the concept of washing of Proban^®^ cotton fabrics at lower pH meets the requirements of functionality and durability, and its acceptability from an ecological point of view requires wastewater treatment using combined methods.

## Figures and Tables

**Table 1:** Characteristics of the cotton fabric.

**Surface Mass (g/m^2^)**	347		
**Density: warp/weft (threads/cm)**	22/34		
**LOI (%)**	32		
**Spectral parameters**	L*	C*	h
**face side**	31.26	40.67	282.37
**back side**	32.88	41.01	281.23
**Digital image, magnification 54×**			
**SEM image, magnification 100×**			

L* – lightness, C* - chroma.

**Table 2:** Description and sample labels.

Proban^®^ Fabric	Description	Washing Bath	Label
Unwashed	Pristine	-	UNW
10 cycles washed	Component dosing in washing process 46	*Det A; *Det B; *A, *B; *NA	46_10x
	Component dosing in washing process 47	Det A; Det B; A, B; NA	47_10x
	Washing process 48	Water (17 ppm)	48_10x
	Washing process 49	*Det ECE-1	49_10x

* Det A: detergent containing fatty alcohol ethoxylate (50–75%), glycol ethers (<20%), amphoteric surfactant (<5%) and propanol (1–5%), A: alkali containing sodium hydroxide (25–35%) and polycarboxylates (<5%), Det B: detergent containing fatty alcohol ethoxylate (<20%), non-ionic surfactants (15–30%), sodium hydroxide (15–20%), sequestering agent (<5%), phosphonates, polycarboxylates (<5%), fluorescent whitening agent, B: disinfecting agent containing hydrogen peroxide (10–20%), peracetic acid (10–20%), acetic acid (25–35%), Det ECE-1: standard detergent according to ISO 105-C06, containing linear alkyl benzene sul-phonate (8%), ethoxylated tallow alcohol, 14 EO (2.9%), sodium soap (3.5%), sodium tripoly-phosphate (43.7%), sodium silicate (7.5%), magnesium silicate (1.9%), carboxymethyl cellulose (1.2%), TAED (0.2%), sodium sulphate (21.2%), water (9.9%), NA: neutralizing agent based on formic acid, p.a.

**Table 3:** Specification of laboratory washing protocols 46, 47, 48 and 49.

Stage	Protocol			
	46	47	48	49
Prewash	Det A: 2.94 g/kg	Det A: 2.94 g/kg	-	-
	60 °C, 5 min			
1st wash	A: 8 mL/kg Det A: 4.90 g/kg Det B: 2.50 g/kg	A: 2.0 mL/kg Det A: 4.90 g/kg Det B: 2.50 g/kg	-	ECE-1: 5 g/kg
	80 °C, 12 min			
2nd wash	A: 5.30 mL/kg Det A: 2.94 g/kg Det B: 2.0 g/kg	- Det A: 2.94 g/kg Det B: 2.0 g/kg	-	ECE-1: 2 g/kg
	80 °C, 1 min			
3rd wash	B: 2.8 mL/kg	B: 2.8 mL/kg	-	-
	80 °C, 20 min			
Cooldown	45 °C, 1 min			
Drain	1 min			
Spin	1 min			
1st rinsing	5 min			
Drain	1 min			
Spin	1 min			
2nd rinsing	3 min			
Drain	1 min			
Spin	1 min			
Neutralization	NA: 3.42 mL/kg	NA: 3.0 mL/kg	-	-
	4 min			
Drain	1 min			
Spin	7 min			

**Table 4:** pH of washing baths.

	46	47	48	49
pre-wash	8.14	8.14	-	-
1st wash	12.28	10.98	-	9.8
2nd wash	12.28	10.98	-	8.6
3rd wash	11.02	7.37	-	-

**Table 5:** Characteristics of washing effluents.

	46	47	48	49
pH	2.82	2.89	5.37	5.42
κ (µS/cm)	790	661	52.1	80
T (NTU)	28.9	30.2	36.8	26.7
TS (mg/L)	1029	936	109	121
TDS (mg/L)	1178	1076	18	60
TSS (mg/L)	66	76	96	55
COD (mg/L)	>1500.00	1318.00	190.33	129.67

**Table 6:** pH of fabrics’ aqueous extract.

Fabric	pH
UNW	7.44
46_10x	7.66
47_10x	7.33
48_10x	8.11
49_10x	7.75

**Table 7:** LOI of fabrics in warp and weft directions.

Sample	LOI (%)
UNW warp/weft	32
46_10x warp/weft	32
47_10x warp/weft	32
48_10x warp/weft	32
49_10x warp/weft	32

**Table 8:** Parameters of MCC analysis unwashed and 10-times-washed Proban^®^ fabric.

Parameters	UNW	46_10x	47_10x	48_10x	49_10x
ηc (J(g·K)^−1^)	61.0	65.7	71.7	62.7	69.3
Qmax (Wg^−1^)	62.5	66.7	72.9	64.0	71.0
hc (kJg^−1^)	2.0	2.1	2.4	1.9	2.1
hc, gas (kJg^−1^)	2.0	2.2	3.8	3.1	3.4
Tmax (°C)	314.1	313.0	314.5	311.0	312.8
Residue (%)	40.81	37.40	35.63	39.83	38.78

**Table 9:** Differences in spectral characteristics of washed samples compared to the pristine sample.

Sample	Side	Mark (ISO)	Spectral Values			
			dE	dL*	dC*	dH*
46_10x	Face	2-3	4.265	4.176	0.820	−0.248
	Back	3	4.024	3.812	1.213	0.137
47_10x	Face	3-4	3.144	2.464	1.876	0.395
	Back	4-5	3.126	0.801	2.685	1.363
48_10x	Face	2-3	4.781	4.732	0.452	−0.344
	Back	3	3.763	3.298	1.747	0.443
49_10x	Face	2-3	5.577	4.99	−1.801	−1.702
	Back	2-3	4.672	4.355	−1.095	−1.286

dE—difference in color, dL*—difference in lightness, dC*—difference in chromaticity, dH*—difference in hue.

**Table 10:** Digital images of samples magnified 54 and 230× and SEM image magnified 500×.

	54×	230×	500×
UNW	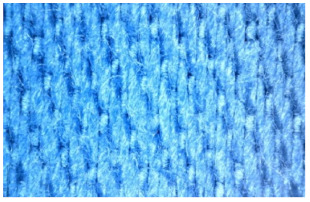	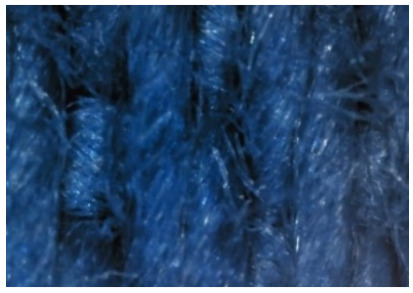	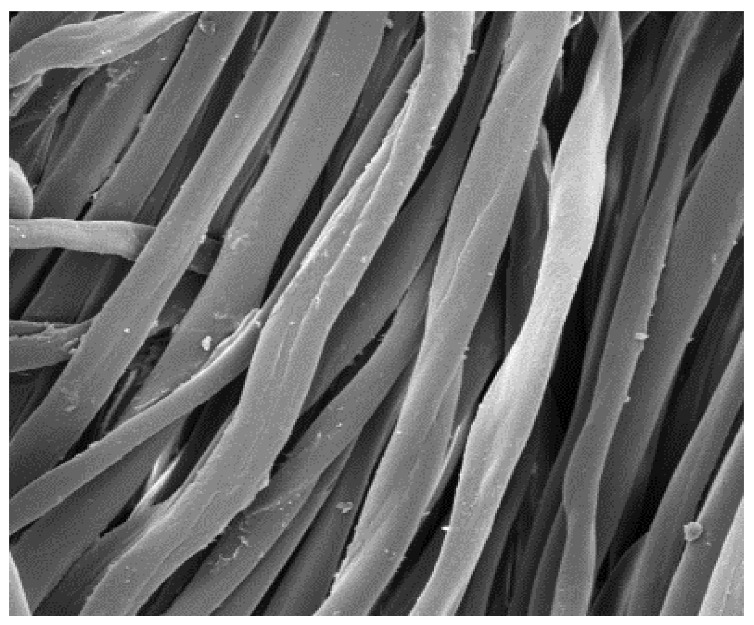
46_10x	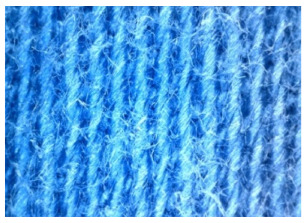	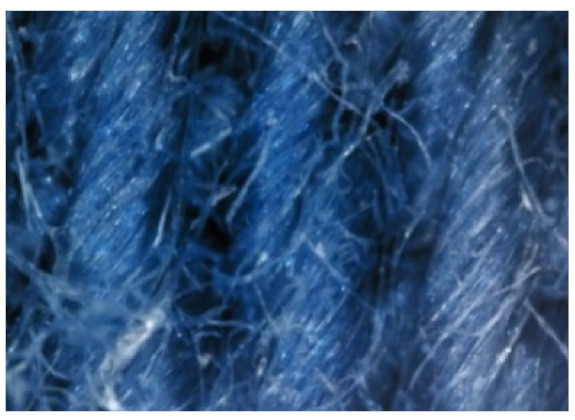	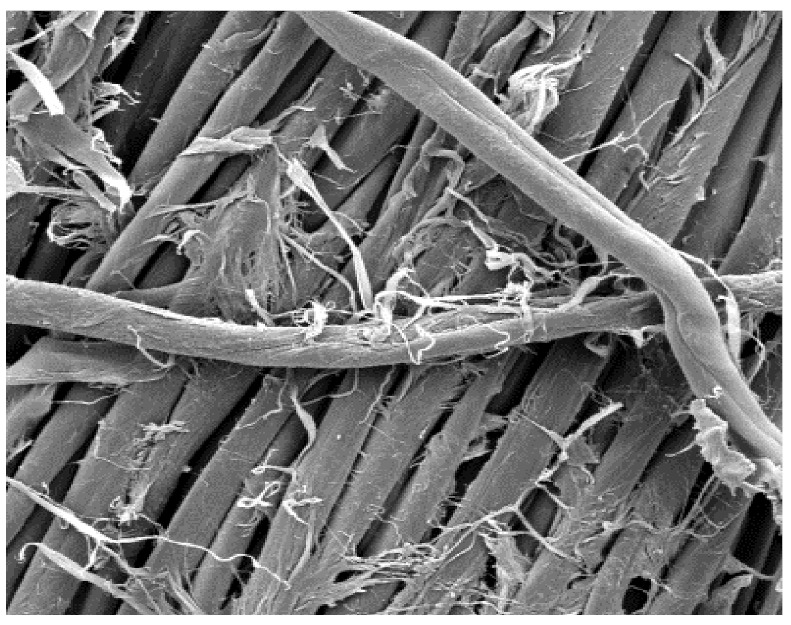
47_10x	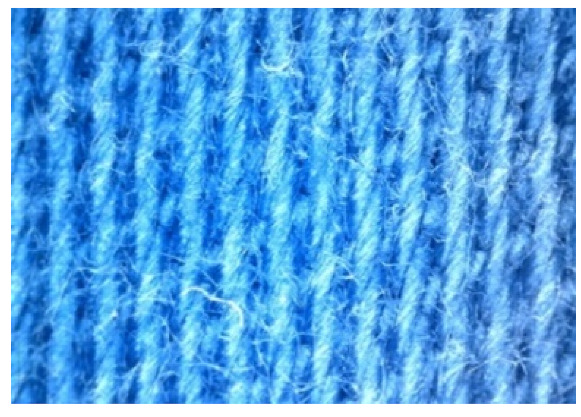	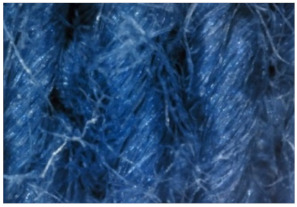	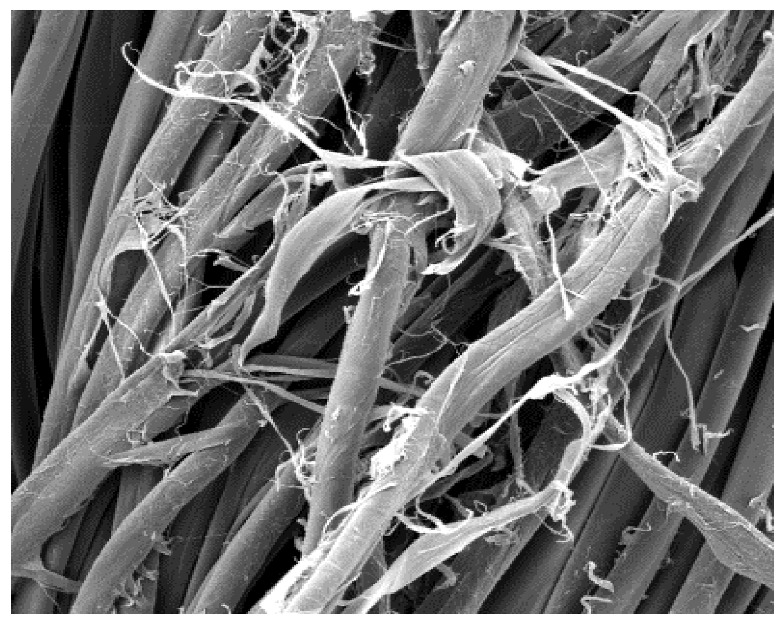
48_10x	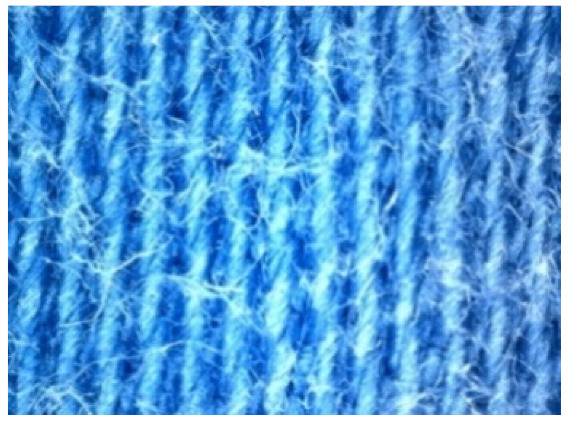	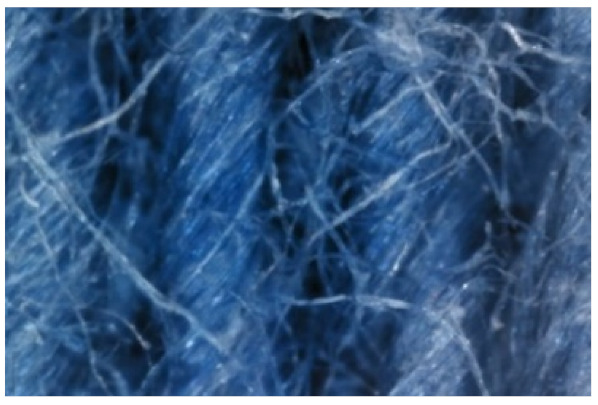	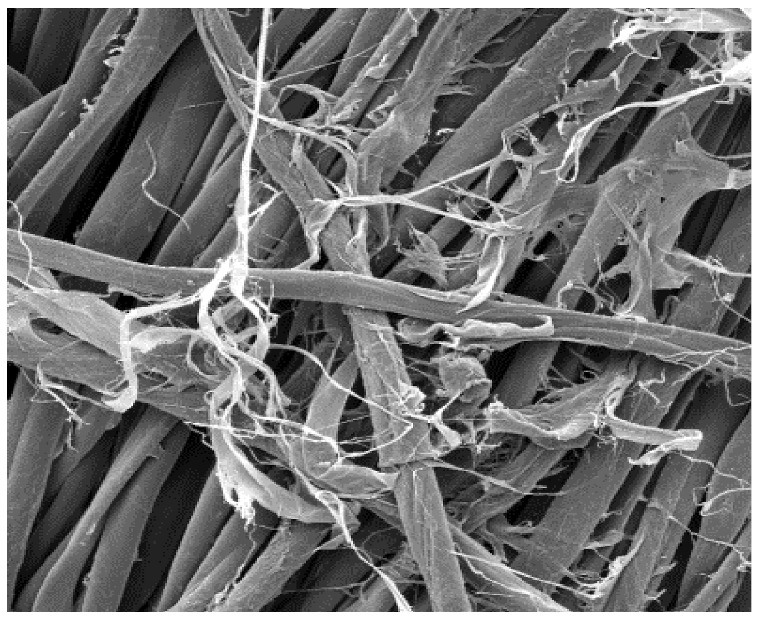
49_10x	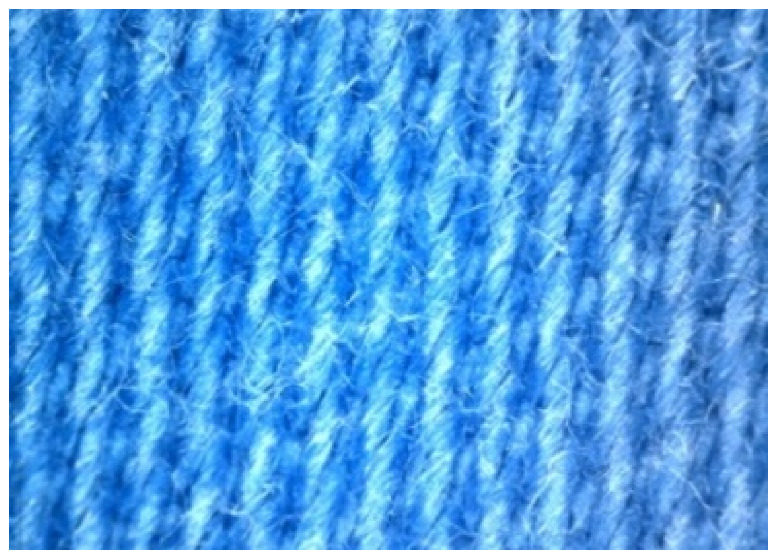	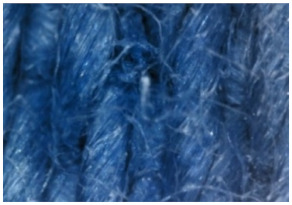	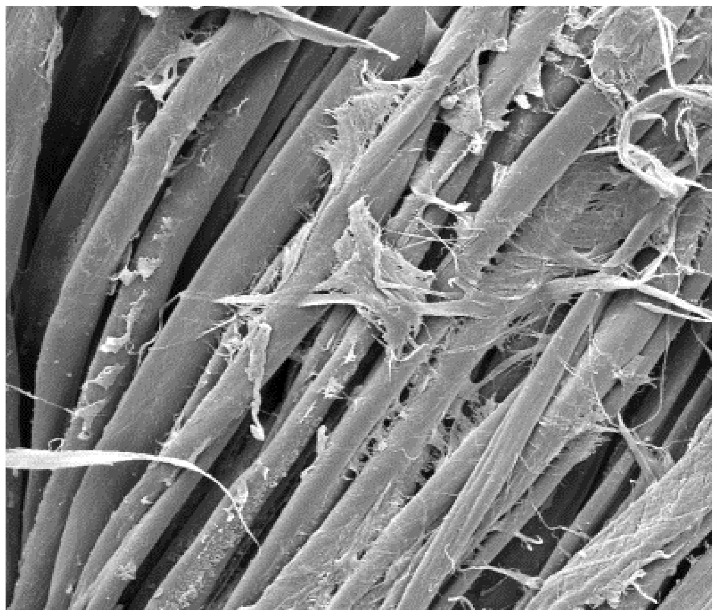

## Data Availability

Data is available in a publicly accessible repository.

## References

[1] Yang C.Q., Weil E.D., Bischof S. (2012). Functional Protective Textiles. Flame Retardant Textiles.

[2] Pavlinić D.Z., House J.R., Mekjavić I.B. (2010). Protupožarni odjevni sustavi i njihovo vrednovanje. Sigurnost.

[3] Chen W.B., Wan Y.Y., Que F., Ding X.M. (2012). The Durability of Flame Retardant and Thermal Protective Cotton Fabrics during Domestic Laundering. Adv. Mater. Res..

[4] House J.R., Squire J.D. (2004). Effectiveness of Proban^®^ flame retardant in used clothing. Int. J. Cloth. Sci. Technol..

[5] Pušić T., Kaurin T. (2017). Zaštitna odjeća Proban^®^ kvalitete—Mogućnosti i rizici. Tekstil.

[6] Alfieri F., Cordella M., Stamminger R., Alexander B. (2018). Durability Assessment of Products: Analysis and Testing of Washing Machines.

[7] Carr C.M. (1995). Chemistry of the Textile Industry.

[8] Alfieri F., Cordella M., Sanfelix J., Dodd N. (2018). An approach to the assessment of durability of energy-related products. Procedia CIRP.

[9] Zhang D., Williams B.L., Liu J., Hou Z., Smith A.T., Nam S., Nasir Z., Patel H., Partyka A., Becher M.A. (2021). An environmentally-friendly sandwich-like structured nanocoating system for wash durable, flame retardant, and hydrophobic cotton fabrics. Cellulose.

[10] Sinner H. (1960). Über das Waschen mit Haushaltswaschmaschinen: In welchem Umfang erleichtern Haushaltwaschmaschinen und -geräte das Wäschehaben im Haushalt.

[11] Boyano A., Cordella M., Espinosa N., Villanueva A., Graulich K., Rüdenauer I., Alborzi F., Hook I., Stamminger R. (2017). Ecodesign and Energy Label for Household Washing Machines and Household Washer-Dryers.

[12] Bao W., Gong R., Ding X., Xue Y., Li P., Fan W. (2017). Optimizing a laundering program for textiles in a front-loading washing machine and saving energy. J. Clean. Prod..

[13] Li C., Wang L., Yuan M., Xu H., Dong J. (2019). A New Route for Indirect Mineralization of Carbon Dioxide–Sodium Oxalate as a Detergent Builder. Sci. Rep..

[14] Nayak R., Ratnapandian S. (2018). Care and Maintenance of Textile Products Including Apparel and Protective Clothing.

[15] Smulders E. (2002). Laundry Detergents.

[16] Flinčec Grgac S., Bischof S., Pušić T., Petrinić I., Luxbacher T. (2017). Analytical Assessment of the Thermal Decomposition of Cotton-Modacryl Knitted Fabrics. Fibres Text. East. Eur..

